# Enhanced survival of BCG-stimulated dendritic cells: involvement of anti-apoptotic proteins and NF-κB

**DOI:** 10.1242/bio.032045

**Published:** 2018-05-30

**Authors:** Pawan Kumar, Vini John, Ananya Gupta, Sangeeta Bhaskar

**Affiliations:** Product Development Cell-I, National Institute of Immunology, Aruna Asaf Ali Marg, New Delhi 110067, India

**Keywords:** BCG, Dendritic cell, Lifespan, Apoptosis, Anti-apoptotic proteins, MyD88, NF-κB

## Abstract

BCG (Bacillus Calmette-Guérin) is the only available vaccine against TB and is also used for the treatment of superficial bladder cancer. BCG-mediated protection against TB and bladder cancer has been shown to rely on its ability to induce superior CD4^+^ and CD8^+^ T cell responses. As the magnitude of T cell responses is defined by dendritic cell (DC) lifespan, we examined the effect of BCG on DC survival and its underlying mechanisms. It was observed that BCG stimulation enhanced DC survival and prolonged DC lifespan in a dose-dependent manner. Live BCG led to a higher DC survival compared with heat-killed BCG. FITC-Annexin V staining showed that BCG promoted DC survival by inhibiting apoptosis. Consistently, higher expressions of anti-apoptotic proteins Bcl-2 and Bcl-x_L_ were observed in BCG-stimulated DCs. Pharmacological inhibition of Bcl-2 and Bcl-x_L_ drastically reduced the DC survival efficacy of BCG. Comparable survival of BCG-stimulated wild-type and MyD88^−/−^ DCs suggested that MyD88 signaling is dispensable for BCG-induced DC survival. NF-κB is one of the key regulators of innate immune responses. We observed that pharmacological inhibition of NF-κB abrogated BCG-mediated increase in DC survival and expression of anti-apoptotic proteins. These findings provide a novel insight into the effect of BCG on DC physiology.

## Abstract (precis)

**Summary:** BCG enhanced the survival of dendritic cells (DCs) and prolonged their lifespan. BCG promoted DC survival by up-regulating Bcl-2 and Bcl-x_L_. Increased survival of BCG-stimulated DCs was dependent on NF-κB, but was independent of MyD88 signaling.

## INTRODUCTION

BCG (Bacillus Calmette-Guérin), which was derived from *Mycobacterium bovi*s nearly 100 years ago, is the only available vaccine against tuberculosis (TB) (Andersen and Doherty, 2005). BCG-mediated protection against TB relies on mounting superior *Mycobacterium tuberculosis* (*Mtb*)-specific CD4^+^ and CD8^+^ T cell responses (Kaufmann, 2006). In vaccinated mice, the immune response against *Mtb* is characterized by an accelerated accumulation of effector T cells at the site of active infection and early production of T_H_1 cytokines, leading to restricted growth of the bacilli (Irwin et al., 2005; Goter-Robinson et al., 2006). BCG is also used for the treatment of superficial bladder cancer. Interestingly, BCG therapy has been found to be more effective than standard chemotherapy, particularly when used against high-grade tumors (Alexandroff et al., 1999). Immunotherapeutic effects of BCG vanish in athymic nude mice, underlining the central importance of T lymphocytes. Both CD4^+^ and CD8^+^ T cells mediate the immunotherapeutic efficacy of BCG as depletion of either cell type results in the failure of BCG therapy (Kawai et al., 2013).

Dendritic cells (DCs), being the most potent antigen-presenting cells, play a key role in mounting T cell responses against *Mtb* and tumor cells. Similar to macrophages, DCs are infected by BCG and other mycobacteria at high frequencies (Wolf et al., 2007). Mycobacteria-infected DCs undergo phenotypic maturation and acquire T cell-activating accessory functions. Studies have shown that, in addition to their maturation status, the lifespan of DCs also plays a pivotal role in defining the magnitude of adaptive immune responses (Hou and Van Parijs, 2004; Nestle, 2006). Increasing DC lifespan by deleting pro-apoptotic genes or by over-expressing anti-apoptotic proteins has been shown to result in heightened T cell responses (Chen et al., 2007a,b). Mechanistically, increased DC lifespan enhances the frequency of productive T cell–DC interactions, leading to the heightened T cell immunity.

Given the protective efficacy of BCG against TB and bladder cancer, and the direct bearing of DC lifespan on the magnitude of T cell responses, here we analyzed the effect of BCG on DC survival and examined its underlying mechanisms. It was observed that BCG enhanced DC survival and prolonged DC lifespan in a dose-dependent manner. BCG-mediated survival of DCs was attributed to reduced apoptosis of these cells. Consistently, higher expression of anti-apoptotic proteins Bcl-2 and Bcl-x_L_ was observed in BCG-stimulated DCs. BCG increased the survival of both wild-type and MyD88^−/−^ DCs. It was further observed that BCG-mediated DC survival was drastically reduced with NF-κB inhibition. These results implicated the roles of anti-apoptotic proteins and NF-κB in BCG-induced DC survival.

## RESULTS

### BCG stimulation enhances DC survival

**Fig. 1.**
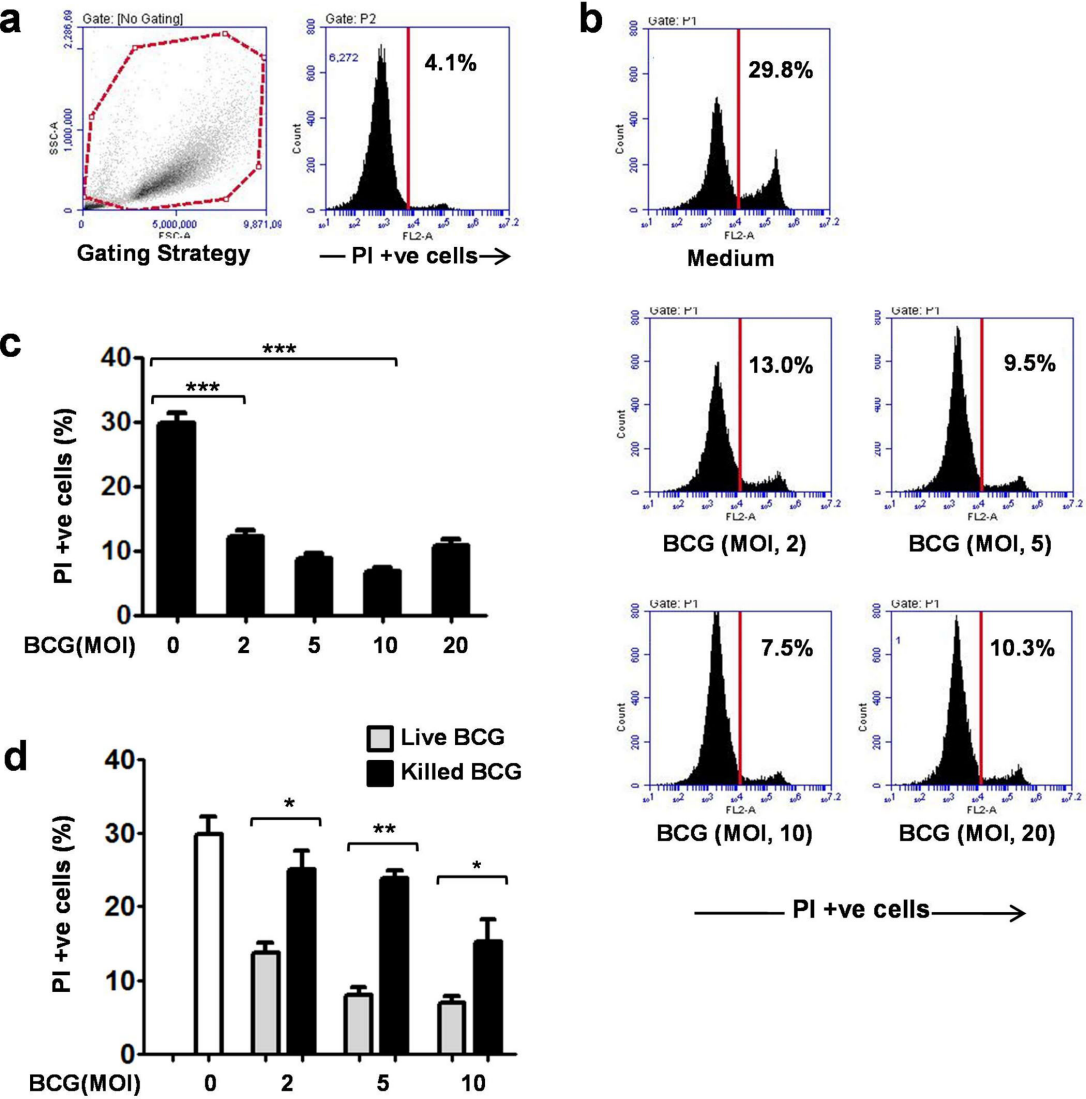
**BCG stimulation enhanced the survival of dendritic cells.** (A) Mouse bone marrow-derived dendritic cells (BMDCs) were harvested on day 7 and analyzed for live/dead cells on the basis of propidium iodide (PI) staining by flow cytometry. Level of PI-positive cells in freshly harvested BMDCs (0 h) was in the range of 3–5%. (B) To analyze the effect of BCG on DC survival, BMDCs were stimulated with BCG at MOI of 2, 5, 10 and 20 for 24 h and analyzed for PI staining. A dose-dependent reduction in the levels of PI-positive cells was observed in BCG-stimulated DCs. Representative data from three independent experiments are shown. (C) Levels of PI-positive cells in unstimulated and BCG-stimulated DCs cultured for 24 h are shown in the form of a bar graph. Mean±s.e.m. of three independent experiments are shown. (D) Live and heat-killed BCG were compared for their DC survival efficacy. Live BCG showed a higher DC survival efficacy, compared with heat-killed BCG. Mean±s.e.m. of three independent experiments are shown. *P<0.05, **P<0.01, ***P<0.001 and ns, not significant (one-way ANOVA).

Mouse bone marrow-derived dendritic cells (BMDCs) were harvested on day 7, and stimulated with BCG at the increasing multiplicity of infection (MOI). After 24 h, the proportion of live/dead cells was analyzed on the basis of propidium iodide (PI) staining by flow cytometry, as described previously (Hou and Van Parijs, 2004; Kumar et al., 2015). It was observed that the level of PI-positive cells in freshly harvested DCs was nearly 4% (Fig. 1A). After 24 h, the level of PI-positive cells in unstimulated DCs reached ∼30%, whereas in BCG-stimulated DC (MOI, 10) it remained 5–7% (Fig. 1B,C). As PI selectively permeates into the nucleus of dead cells, a decreased proportion of PI-positive cells in BCG-stimulated DCs demonstrated their enhanced survival. Similar to our findings, a high level of cell death in unstimulated DCs has been reported previously (Hou and Van Parijs, 2004; Kumar et al., 2015). We further observed that DC survival was enhanced with the increasing MOI of BCG, but was compromised at the MOI of 20, probably due to excessive bacillary burden (Fig. 1B,C).

Earlier studies have shown that live BCG confers higher protection against TB, compared with killed BCG (Chen et al., 2003). To examine whether this trend is also reflected in DC survival efficacy of BCG, BMDCs were stimulated with live and heat-killed BCG and analyzed for PI staining. It was observed that live BCG led to a significantly higher DC survival, compared with heat-killed BCG (Fig. 1D).

### BCG-stimulated DCs have prolonged lifespan

**Fig. 2.**
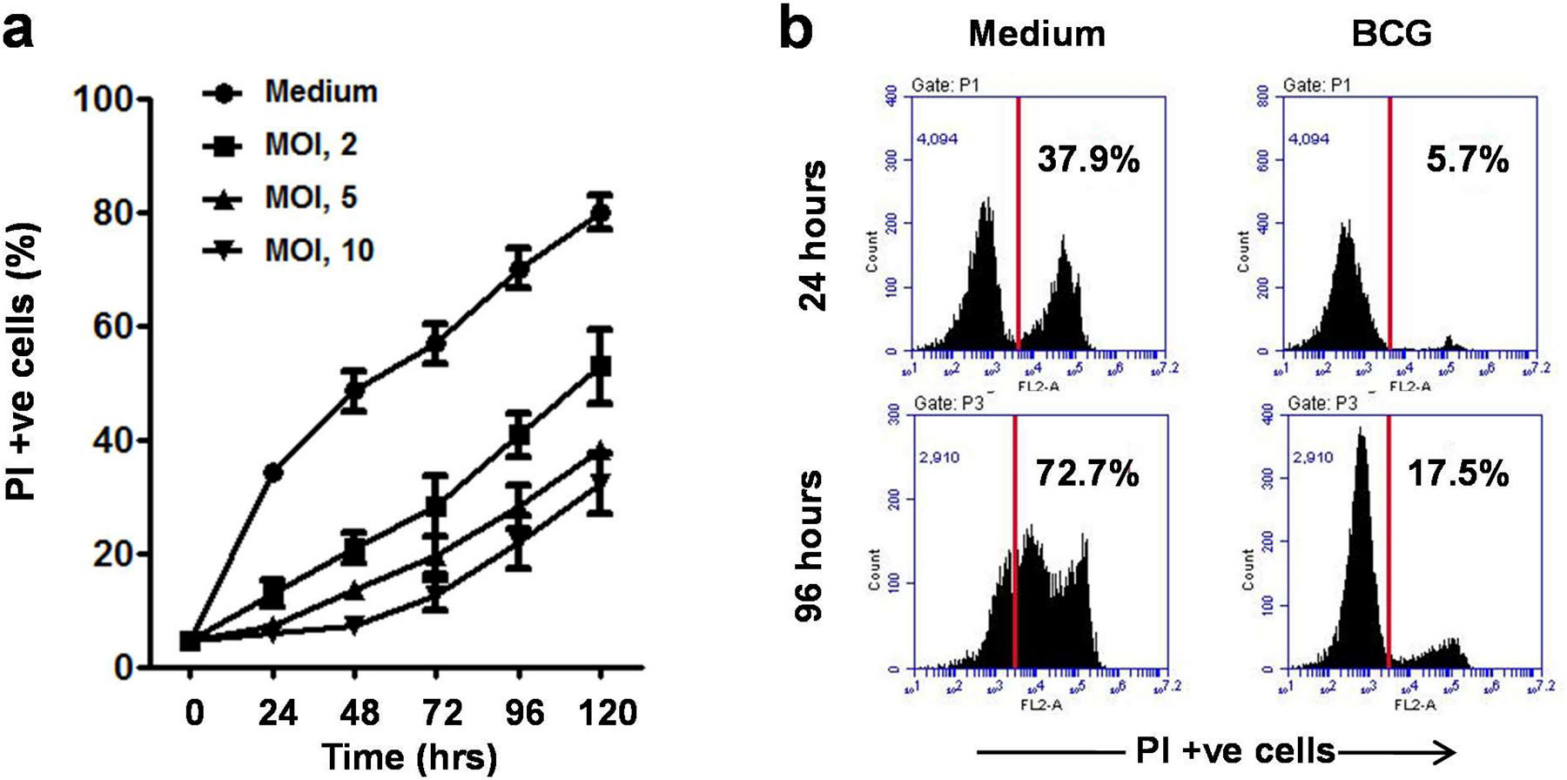
**BCG prolonged the lifespan of dendritic cells.** (A) Mouse BMDCs were stimulated with BCG at MOI of 2, 5 and 10 and analyzed for PI staining at 24 h intervals. Enhanced survival of BCG-stimulated DCs was noted till observation period of 120 h. Mean±s.e.m. of four independent experiments are shown. (B) Percentages of PI-positive cells in BCG-stimulated and unstimulated DCs at 24 h and 96 hours' time points are shown in histograms. Representative data from four independent experiments are shown.

The above experiments analyzed the DC survival at 24 h time-point. To examine the effect of BCG on DC lifespan, time-kinetics studies were undertaken. BCG-stimulated DCs were analyzed for PI staining at 24 h intervals. It was observed that BCG enhanced DC survival till the observation period of 120 h (Fig. 2A). At 96 h, the level of PI-positive cells in unstimulated DCs was 72.7%, whereas in BCG-stimulated DCs (MOI, 10), it was 17.5% (Fig. 2B). It was further observed that BCG prolonged DC lifespan in a dose-dependent manner and maximum longevity was noticeable at MOI of 10.

### BCG promotes DC survival by inhibiting apoptosis

**Fig. 3.**
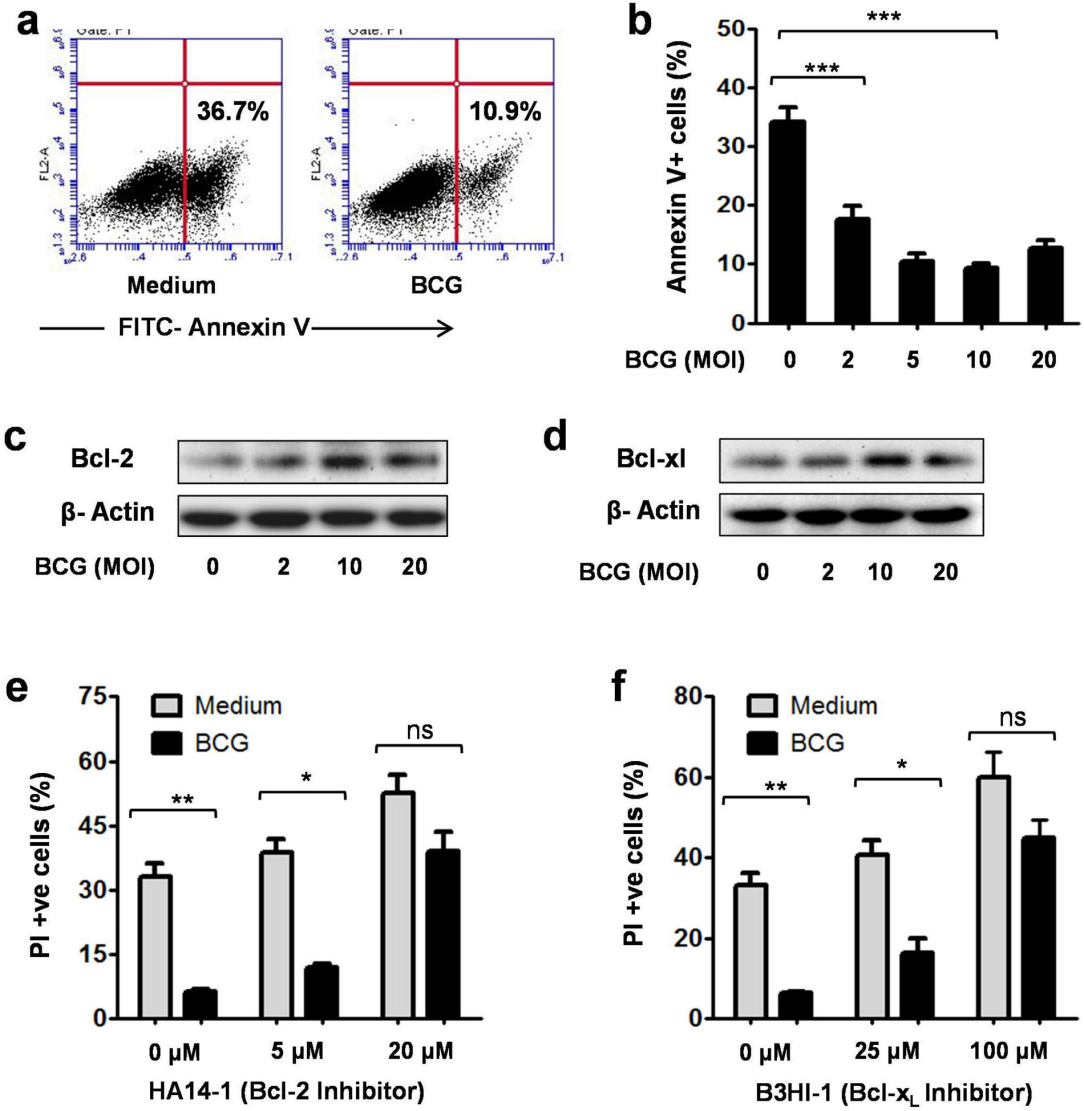
**BCG promoted the DC survival by inhibiting apoptosis.** (A) BMDCs were stimulated with BCG for 24 h and analyzed for FITC-annexin V staining by flow cytometry as suggested by the manufacturer (BD Biosciences). Reduced levels of annexin V^+^ cells were observed in BCG (MOI,10)-stimulated DCs. Representative data from three independent experiments are shown. (B) BCG decreased the levels of Annexin V^+^ cells in a dose-dependent manner. Mean±s.e.m. of three independent experiments are shown. (C,D) Cellular lysates prepared from unstimulated and BCG-stimulated DCs were analyzed for Bcl-2 and Bcl-x_L_ by immunoblotting. Higher expression of Bcl-2 and Bcl-x_L_ was observed in BCG-stimulated DCs, compared with unstimulated DCs. BCG induced the maximal expression of these proteins at the MOI of 10. Representative data from two independent experiments are shown. (E,F) DCs were stimulated with BCG (MOI, 10) in presence of specific inhibitors of Bcl-2 and Bcl-x_L_ and analyzed for PI staining after 24 h. Inhibition of Bcl-2 and Bcl-x_L_ significantly increased the levels of PI-positive cells in BCG-stimulated DCs. Mean±s.e.m. of three independent experiments are shown. *P<0.05, **P<0.01, ***P<0.001 and ns, not significant (one-way ANOVA).

Similar to other leukocytes, DCs are wired to undergo apoptosis when their defined function or lifespan is over (Kamath et al., 2000). We hypothesized that inhibition of DC apoptosis could be one possible mechanism for enhanced survival of BCG-stimulated DCs. To examine this, BCG-stimulated DCs were analyzed for FITC-annexin V staining by flow cytometry. Annexin V binds to phosphatidylserine, which accumulates in the outer leaflet of an early apoptotic cell's plasma membrane. We observed significantly reduced levels of FITC-annexin V^+^ cells in BCG-stimulated DCs, compared with unstimulated DCs (Fig. 3A,B). In unstimulated DCs, 36.7% of cells were annexin V^+^, whereas in BCG-stimulated DCs (MOI, 10), the level of annexin V^+^ cells was 10.9%. Consistent with above results, BCG inhibited DC apoptosis in a dose-dependent manner (Fig. 3B).

Since anti-apoptotic proteins are key regulators of apoptotic cell death, their role in BCG-induced DC survival was analyzed. Cellular lysates prepared from unstimulated and BCG-stimulated DCs were probed for Bcl-2 and Bcl-x_L_ by immunoblotting. It was observed that BCG-stimulated DCs expressed higher levels of Bcl-2 and Bcl-x_L_, compared with unstimulated DCs (Fig. 3C,D). Maximum expression of these proteins was observed at the MOI of 10. The roles of Bcl-2 and Bcl-x_L_ in BCG-induced DC survival were further confirmed with help of their pharmacological inhibitors. Inhibition of Bcl-2 resulted in significantly reduced survival of BCG-stimulated DCs (Fig. 3E). Similarly, BCG-induced DC survival was significantly decreased with inhibition of Bcl-x_L_ (Fig. 3F).

### MyD88 signaling is dispensable for BCG-induced DC survival

**Fig. 4.**
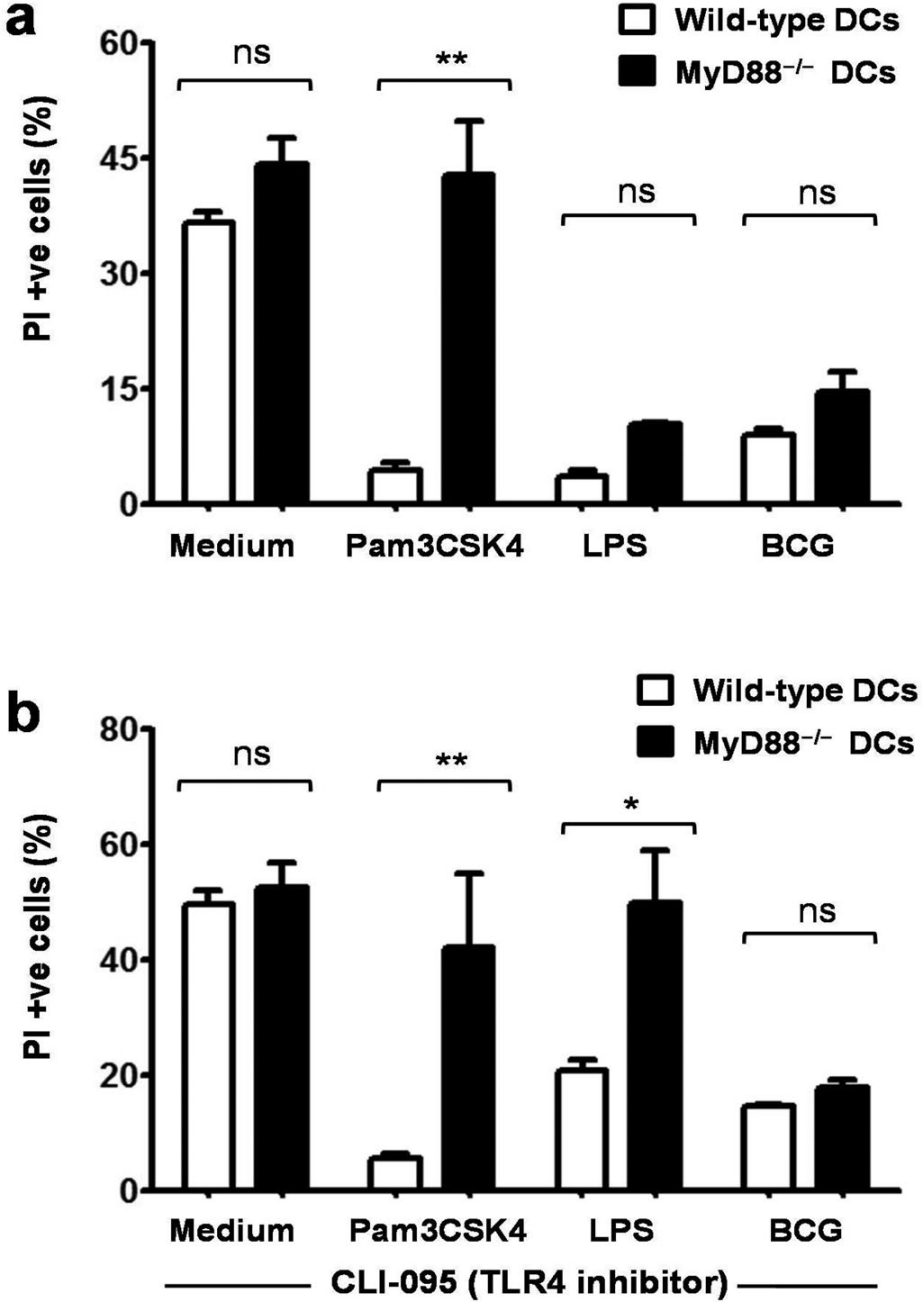
**MyD88 signaling is dispensable for BCG-induced dendritic cell survival.** (A) Wild-type and MyD88^−/−^ DCs were stimulated with BCG (MOI, 10) for 24 h and analyzed for PI staining by flow cytometry. Comparable levels of PI-positive cells were observed in BCG-stimulated wild-type and MyD88^−/−^ DCs. Pam3CSK4 (a synthetic TLR2 ligand) and LPS (TLR4 ligand) were used as controls. Mean±s.e.m. of three independent experiments are shown. (B) To examine the putative role of MyD88-independent TLR4 signaling in BCG-induced DC survival, DCs were stimulated with BCG in the presence of TLR4 signaling inhibitor (CLI-095). No significant difference in the levels of PI-positive cells was observed in DCs stimulated with BCG in the presence or absence of CLI-095. Mean±s.e.m. of three independent experiments are shown. *P<0.05, **P<0.01 and ns, not significant (one-way ANOVA).

MyD88 is an adaptor protein involved in toll-like receptor (TLR)-mediated recognition of BCG and other mycobacteria (Barton and Medzhitov, 2003). Previous studies have shown that MyD88-deficiency affects some aspects (viz. cytokine secretion) of DC functions while others remain unaffected (Fremond et al., 2004). To examine the role of MyD88 in BCG-induced DC survival, wild-type and MyD88^−/−^ DCs were stimulated with BCG and analyzed for PI staining. Interestingly, we observed that BCG enhanced the survival of both wild-type and MyD88^−/−^ DCs to the comparable levels (Fig. 4A). Pam3CSK4 (a synthetic TLR2 ligand) and LPS (TLR4 ligand) were used as controls. The effect of Pam3CSK4 on DC survival was drastically reduced in MyD88^−/−^ DCs, but as in the case of BCG, LPS led to the comparable survival of wild-type and MyD88^−/−^ DCs. Since TLR4 can act in both MyD88-dependent and -independent manners (Kawai and Akira, 2010), LPS-induced survival of knockout DCs was attributable to MyD88-independent TLR4 signaling.

Given the TLR4 agonist activity of BCG (Heldwein et al., 2003), we examined the role of MyD88-independent TLR4 pathway in BCG-induced DC survival. Wild-type and MyD88^−/−^ DCs were stimulated with BCG in the presence of the TLR4 signaling inhibitor CLI-095 and analyzed for PI staining by flow cytometry. We observed that inhibition of TLR4 signaling did not preclude BCG-induced survival of wild-type or MyD88^−/−^ DCs (Fig. 4B). These findings showed that MyD88-dependent or -independent TLR signaling is dispensable for BCG-induced DC survival.

### NF-κB plays a critical role in enhanced survival of BCG-stimulated DCs

**Fig. 5.**
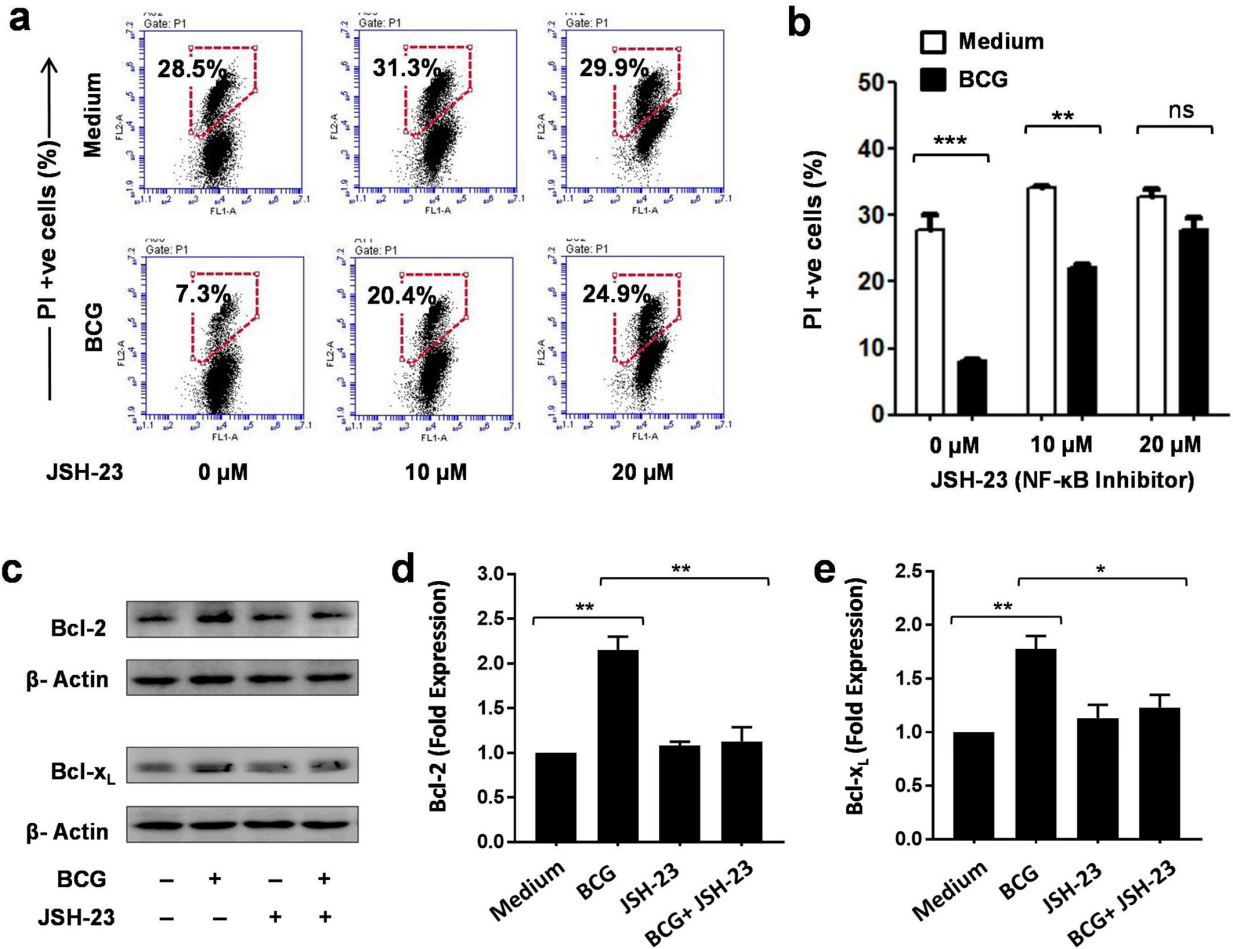
**BCG enhanced dendritic cell survival in NF-κB-dependent manner.** DCs were stimulated with BCG in the presence of NF-κB inhibitor JSH-23 for 24 h and analyzed for PI staining by flow cytometry. (A) JSH-23 led to a dose-dependent increase in levels of PI-positive cells in BCG-stimulated DCs. Representative data from three independent experiments are shown. (B) Bar graph representation of PI-positive cells in BCG-stimulated DCs cultured in the presence of an increasing concentration of JSH-23. Mean±s.e.m. of three independent experiments are shown. (C) Cellular lysates from DCs stimulated in the presence of JSH-23 were analyzed for the expression of anti-apoptotic proteins by immunoblotting. Comparable levels of Bcl-2 and Bcl-x_L_ were observed in JSH-23-treated, unstimulated and BCG-stimulated DCs. (D,E) Bar graphs of Bcl-2 and Bcl-x_L_ levels in dendritic cells stimulated with BCG in the presence or absence of JSH-23. Mean±s.e.m. of three independent experiments are shown. *P<0.05, **P<0.01, ***P<0.001 and ns, not significant (one-way ANOVA).

NF-κB is one of the key regulators of innate immune responses to a variety of microbial stimuli (Hayden et al., 2006). BCG and other mycobacteria have been shown to induce cytokine secretion in DCs and other immune cells in an NF-κB-dependent manner (Zhang et al., 2013). We asked if NF-κB is also involved in the enhanced survival of BCG-stimulated DCs. To examine this, DCs were stimulated with BCG in the presence of JSH-23 (a specific inhibitor of NF-κB) and analyzed for PI staining. It was observed that NF-κB inhibition with JSH-23 abrogated BCG-induced DC survival in a dose-dependent manner (Fig. 5A,B). At 20 µM concentration, it resulted in comparable levels of PI-positive cells in unstimulated and BCG-stimulated DCs.

Since anti-apoptotic proteins were found to play a key role in BCG-mediated DC survival, we asked whether expression of these proteins in BCG-stimulated DCs depends on NF-κB. To examine this, cellular lysates from JSH-23-treated, BCG-stimulated DCs were probed for Bcl-2 and Bcl-x_L_. Consistent with earlier observations, enhanced expression of Bcl-2 and Bcl-x_L_ was observed in BCG-stimulated DCs, compared with unstimulated DCs. Interestingly, inhibition of NF-κB markedly reduced the expression of anti-apoptotic proteins in BCG-stimulated DCs (Fig. 5C,D,E). These results showed that BCG promoted DC survival by up-regulating Bcl-2 and Bcl-x_L_ expression in an NF-κB-dependent manner.

## DISCUSSION

Cellular physiology of DCs plays a critical role in regulating the nature and intensity of adaptive immune responses. Largely, the intensity of T cell response is defined by DC lifespan (Kushwah and Hu, 2010). With increased lifespan, antigen-loaded DCs can interact with a higher proportion of cognate T cells, resulting in the heightened T cells responses. Efficacy of BCG against TB and bladder cancer immunotherapy has been shown to rely on its ability to induce superior T cell responses (Kaufmann, 2006; Kawai et al., 2013). Since DC lifespan has a direct bearing on the magnitude of immune response, we wondered how BCG affects DC survival. We observed that BCG promoted DC survival in a dose-dependent manner, with optimal survival observed at MOI of 10. These results were also reflected in the prolonged lifespan of BCG-stimulated DCs. In 4 days (120 h) culture, level of PI-positive cells in BCG-stimulated DCs was nearly one-fourth of PI-positive cells in unstimulated DCs.

The effect of other microorganisms on DC survival has been studied previously. It has been reported that Gram-positive bacteria induce DC apoptosis via bacteria-encoded virulence factors (Nogueira et al., 2009). Similarly, many Gram-negative bacteria have been shown to promote DC apoptosis by activating caspase-3 or caspase-8 (Gröbner et al., 2007). Interestingly, *Mycobacterium tuberculosis* (*Mtb*) has also been shown to result in the enhanced killing of DCs (Ryan et al., 2011). A role of ESX-1 (ESAT-6 secretion system-1) has been implicated in the *Mtb*-mediated killing of DCs. Consistent with the opposing effects of BCG and *Mtb* on DC survival, the ESX-1 system has been reported missing from BCG (Brodin et al., 2006).

BCG vaccine is used only in live form. In fact, live BCG has been shown to confer significantly higher protection against TB, compared with heat-killed BCG. In keeping with these observations, our results showed a significantly enhanced survival of live BCG-stimulated DCs, compared with heat-killed BCG-stimulated DCs. In our previous studies with an atypical mycobacterium species, *Mycobacterium indicus pranii*, we have observed that potential immunostimulatory molecules are obscured in the heat-killed form of bacilli (Kumar et al., 2014). It is probable that similar mechanisms lead to lower DC survival efficacy of heat-killed BCG.

Next, we sought to investigate how BCG exerts its cell survival effect on DCs. For apoptosis is a key homeostatic phenomenon involved in the disposal of immune cells, we examined the effect of BCG on DC apoptosis. Our results showed that BCG inhibits DC apoptosis in a dose-dependent manner. An important aspect of reduced DC apoptosis is that it would allow some bacilli to persist inside the host body for longer duration (Fairbairn, 2004). Since anti-TB efficacy of BCG is dependent on its persistence inside the host body (Brandt et al., 2002), reduced apoptosis of infected DCs might be contributing to the protective efficacy of BCG. Consistent with their reduced apoptosis, we observed higher expressions of anti-apoptotic proteins Bcl-2 and Bcl-x_L_ in BCG-stimulated DCs. Inhibition of Bcl-2 or Bcl-x_L_ resulted in the comparable levels of PI-positive cells in unstimulated and BCG-stimulated DCs. The roles of Bcl-2 and Bcl-x_L_ in promoting DC survival has also been demonstrated with LPS-stimulated DCs (Hou and Van Parijs, 2004).

Innate immune cells sense invading microorganisms with the help of pattern recognition receptor (PRRs). TLRs are among most prominent and most studied PRRs and employ MyD88 adaptor protein for trans-membrane passage of microbial signals (Barton and Medzhitov, 2003). Therefore, we asked whether BCG promotes DC survival in MyD88-dependent manner. Surprisingly, we observed that BCG-induced the comparable survivals in wild-type and MyD88-deficient DCs. These results showed that MyD88 signaling is dispensable for BCG-mediated DC survival. It is likely that in the absence of MyD88, some other PRRs contribute to mycobacterial recognition by DCs. Supporting this, MyD88-deficient DCs have been shown to produce nitrite and to upregulate expression of co-stimulatory molecules in response to different mycobacterial species (Fremond et al., 2004).

NF-κB, which belongs to a category of fast-acting transcription factors, is the key regulator of innate immune responses (Hayden et al., 2006). Previous studies have shown the role of NF-κB in cytokine production by BCG-stimulated macrophages and DCs (Darieva et al., 2000). We observed drastic reduction in the DC survival efficacy of BCG in the presence of NF-κB inhibitor, suggesting that NF-κB plays an important role in promoting DC survival in response to BCG. Similar to our findings, previous studies have shown that CD40- and TRANCE-induced DC survival is precluded in p50^−/−^ cRel^−/−^ DCs (Ouaaz et al., 2002). Interestingly, inhibition of NF-κB also abrogated the expression of Bcl-2 and Bcl-x_L_ in BCG-stimulated DCs. These findings suggested that in BCG-stimulated DCs, NF-κB promotes cell survival by upregulating the expression of anti-apoptotic proteins. NF-κB has been shown to regulate the transcription of genes encoding Bcl-2 and Bcl-x_L_ in other cell types such as cancer cells and CD4^+^ T lymphocytes (Catz and Johnson, 2001; Chen et al., 2000; Khoshnan et al., 2000).

How BCG activates NF-κB in DCs is an important question arising from this study. DCs express a larger repertoire of PRRs, which may show functional redundancy or may interact with each other in a cooperative/synergistic manner. Functional redundancy of PRRs can be seen in NF-κB activation, wherein most of the PRR signaling pathways converge. Therefore, it is likely that in the absence of TLR/MyD88, other PRRs (e.g. DC-SIGN, CLR) activates NF-κB and promotes DC survival. Interestingly, owing to their complex cell wall, mycobacteria can engage a variety of PRRs. We are further examining the role of different PRRs in the enhanced survival of BCG-stimulated DCs.

In conclusion, our findings established the enhanced survival of BCG-stimulated DCs and delineated its underlying mechanism. Enhanced DC survival has been shown to result in heightened T cell responses. In the case of BCG, it could be a novel mechanism contributing to its protective efficacy against TB and immunotherapeutic effects against bladder cancer.

## MATERIALS AND METHODS

### Animals and ethics statement

Inbred, 6–8-week-old C57BL/6 and MyD88^−/−^ male mice were obtained from the Small Animal Facility of the National Institute of Immunology, New Delhi, India. All animal experiments were approved by the institutional animal ethics committee (IAEC) of the National Institute of Immunology, New Delhi, India and were performed in accordance with the guidelines of the same (IAEC approval no. 205/08/13).

### BCG culture and preparation

BCG was cultured in Middlebrook 7H9 broth medium (BD Difco) having 0.05% tween-80, 0.05% glycerol and 10% albumin-dextrose-catalase (ADC) supplement. Log-phase cultures were harvested by centrifugation at 1000 ***g*** for 10 min, and were washed with PBS having 3% FBS (PBS-3). Bacterial aggregates were removed by additional centrifugation at 50 ***g*** for 10 min. The bacillary count was determined on the basis of optical density at 600 nm. Heat-killed BCG was prepared by autoclaving bacterial suspension at 15 psi for 15 min.

### Bone marrow-derived dendritic cells (BMDCs)

BMDCs were prepared by culturing mouse bone marrow cells in the presence of GM-CSF as described previously (Inaba et al., 1992). Briefly, 4×10^6^ bone-marrow cells were added per well of a six-well plate in RPMI-10 medium (RPMI base medium having 10% FBS and 1% penicillin-streptomycin solution) supplemented with 20 ng/ml GM-CSF (PeproTech, Rehovot, Israel). Culture medium along with non-adherent cells were removed on day 3 and day 5, and fresh GM-CSF-supplemented medium was added to each well. Immature DCs were harvested on day 7 by gentle pipetting. After giving a wash with RPMI-10 medium, these cells were used for subsequent experiments.

### DC stimulation with BCG

Wild-type or MyD88^−/−^ DCs were added in a 24-well plate (1.5×10^6^ cells per well). BCG was added to cultures at the indicated multiplicity of infection (MOI). Cells were harvested after 24 h and analyzed for Propidium Iodide (PI) or Annexin V staining. For time-kinetics study, DCs were analyzed for PI staining at 24 h interval till 120 h.

### FITC-annexin V and propidium iodide (PI) staining

For FITC-annexin V staining, 1.0×10^6^ cells were suspended in 100 µl annexin staining buffer and 5 µl FITC-Annexin V solution (BD Biosciences) was added to the suspension. After 15 min, the volume of suspension was made up to 500 µl with annexin staining buffer. For PI staining, 5 µl of 100 µg/ml PI solution was added to DC suspensions. Cells were immediately analyzed by flow cytometry.

### Flow cytometry

BCG-stimulated DCs were harvested, stained with FITC-annexin V and/or PI and acquired on BD Accuri C6 flow cytometer. Data were analyzed with BD Accuri C6 software.

### Immunoblotting

Cellular lysates of unstimulated and BCG-stimulated DCs were prepared with the help of a M2 lysis buffer. Protein concentration in lysates was determined with Pierce BCA protein assay kit. 10–15 µg protein was loaded per well in 12% polyacrylamide gel and resolved at 30 mA. Subsequently, proteins were transferred onto 0.2 µm PVDF membrane at 120 mA for 2 h. Membranes were blocked overnight with 1% bovine serum albumin in Tris-buffered saline (TBS) at 4°C and thereafter, probed with anti-Bcl-2 (1:1000), anti- Bcl-x_L_ (1:2000) and anti-beta-actin (1:10,000) antibodies (Cell Signaling Technology, cat no. 2876, 2764 and 8457, respectively). After washing with TBST, membranes were incubated with secondary antibodies at 25°C for 2 h. After repeated washing, blots were developed using ECL chemiluminescent substrate according to instructions given by the manufacturer (Bio-Rad) and images were acquired using ChemiDoc Imaging System. Densitometry was performed using ImageJ software (NIH).

### Inhibition of Bcl-2, Bcl-x_L_, TLR4, and NF-κB

Inhibitors of Bcl-2 (HA14-1), Bcl-x_L_ (B3HI-1), TLR4 (CLI-095), and NF-κB (JSH-23) were added to DC cultures at indicated concentrations. After 1 h, BCG was added to the cultures and plates were kept in the CO_2_ incubator. DCs were harvested after 24 h and analyzed for PI staining by flow cytometry.

### Statistical analysis

Statistical analyses were performed with the help of GraphPad Prism 5.0 Software. Data were analyzed by one-way analysis of variance (ANOVA) with Tukey's multiple comparison test applied post analysis. *P* values of <0.05 were considered significant.
